# A combined experimental-computational approach for spatial protection efficacy assessment of controlled release devices against mosquitoes (*Anopheles*)

**DOI:** 10.1371/journal.pntd.0007188

**Published:** 2019-03-11

**Authors:** Ulrich R. Bernier, Daniel L. Kline, Agustin Vazquez-Abad, Melynda Perry, Lee W. Cohnstaedt, Pablo Gurman, Sebastián D’hers, Noel M. Elman

**Affiliations:** 1 United States Department of Agriculture-Agricultural Research Service, Center for Medical, Agricultural, and Veterinary Entomology, Gainesville, Florida, United States of America; 2 Instituto Tecnológico de Buenos Aires (ITBA), Ciudad Autónoma de Buenos Aires, Argentina; 3 Textile Materials Evaluation Team, The US Army Natick Soldier Research, And Development Engineering Center (NSRDEC), Natick, MA, United States of America; 4 United States Department of Agriculture-Agricultural Research Service, The Arthropod-Borne Animal Diseases Research Unit (ABADRU), Manhattan, KS, United States of America; 5 GearJump Technologies LLC, Brookline, MA, United States of America; Imperial College London, UNITED KINGDOM

## Abstract

This work describes the use of entomological studies combined with *in silico* models (computer simulations derived from numerical models) to assess the efficacy of a novel device for controlled release of spatial repellents. Controlled Release Devices (CRDs) were tested with different concentrations of metofluthrin and tested against *An*. *quadrimaculatus* mosquitoes using arm-in cage, semi-field, and outdoor studies. Arm-in-cage trials showed an approximate mean values for mosquito knockdown of 40% and mosquito bite reduction of 80% for the optimal metofluthrin formulation for a 15-minute trial. Semi-field outdoor studies showed a mean mortality of a 50% for 24 hour trial and 75% for a 48 hour trial for optimal concentrations. Outdoors studies showed an approximate mean mortality rate of 50% for a 24 hour trial for optimal concentrations. Numerical simulations based on Computational Fluid Dynamics (CFD) were performed in order to obtain spatial concentration profiles for 24 hour and 48 hour periods. Experimental results were correlated with simulation results in order to obtain a functional model that linked mosquito mortality with the estimated spatial concentration for a given period of time. Such correlation provides a powerful insight in predicting the effectiveness of the CRDs as a vector-control tool. While CRDs represent an alternative to current spatial repellent delivery methods, such as coils, candles, electric repellents, and passive emanators based on impregnated strips, the presented method can be applied to any spatial vector control treatment by correlating entomological endpoints, i.e. mortality, with in-silico simulations to predict overall efficacy. The presented work therefore presents a new methodology for improving design, development and deployment of vector-control tools to reduce transmission of vector-borne diseases, including malaria and dengue.

## Author summary

Spatial Repellents (SRs) represent another tool to fight vector-borne diseases, such as malaria and dengue. Newly developed active ingredients were designed to repel or kill vectors in space, creating a shield effect, unlike topical repellents, such as DEET, that rely on vectors to be near or in physical contact with protected target. Metofluthrin and transfluthrin are examples of active ingredients (AIs) designed as SRs against mosquitoes. The efficacy of SRs heavily depends on the delivery method. Currently, there is a lack of fundamental understanding of effectiveness of SR delivery methods. Current delivery modalities of SR do not rely on quantitative models to estimate targeted efficacy, making the end-user overshoot or undershoot the dosage required for protection. Optimizing the dosage over time is critical to obtain protection for a given space in a giving period of time, as well to prevent AI resistance in the long run. The key is therefore to deliver just enough dosage needed to repel or kill the vectors. The presented work provides a novel approach to predict performance of SRs based on an experimental-computational methodology to quantify effectiveness of controlled release devices as a function of AI physical properties (e.g. volatility), device design parameters combined with physical variables, and environmental conditions (e.g. temperature, wind velocity). This work therefore provides the groundwork for estimating quantitative effectiveness of SR delivery methods against mosquitoes.

## Introduction

Vector-borne diseases represent a global public health threat. More than 1 million people die annually of vector-borne diseases. Malaria alone is responsible for 400,000 deaths a year, and most cases are children under five years of age [1–3]. Current vector control techniques can be divided into contact repellent and spatial repellent products [4–7]. Current strategies against mosquitoes include products applied to the skin, insecticide treated nets (ITNs) and indoor residual spraying (IRS). Some topical repellents suffer from undesired odor and texture, resulting in poor user acceptability. Topical repellents presence lowers in time due to skin washing, sweating, clothe rubbing or skin absorption, requiring several applications per day, and thus are limited in duration and rely on user compliance [8,9]. ITNs and IRS are inexpensive, effective indoors and when individuals remain in the confines of the treated areas (building), however neither are an option for outdoors or active users outside of the residence. [10].

Spatial repellent products include electrical repellents, passive emanators based on impregnated strips, candles, and, coils [11]. Electrical repellents, including motorized fans, require power to operate, making their widespread use limited. In addition, many of these devices require frequent cartridge replacement. Passive emanators using impregnated strips are limited to a single compound showing limited efficacy only for short periods of time [12,13]. Candles are based on essential oils, whose efficacy has been questioned [14]. Furthermore, candles and coils require an open flame or ember to operate, representing a fire hazard, and are limited in duration, requiring frequent replacement. Coils are the most widely used products for low income countries, where electricity is not widespread. Currently, the number of coils sold worldwide is estimated at 30 billion yearly at estimated price range of US$ 0.025–0.1 per unit [15]. Their competitive cost together with their ease of use provides compelling reasons to be considered as one of the most widely accepted consumer products for vector control. Coils exhibit a number of disadvantages, including: the Active Ingredient (AI) represents less than 1% of the overall device volume or mass; it requires an open flame starter; it is a fire hazard during operation; it poses respiratory-related health risks, and it lacks continuous release that limits their use to 4–12 hours per unit [16,17].

In addition to the available armamentarium of spatial repellent devices, active devices based on Micro-Electro-Mechanical Systems (MEMS), have been reported as a potential tool against mosquitoes [18]. In spite of their remarkable advantages, including low cost, batch fabrication, small form factor and the ability to actively control the delivery process, MEMS require power to operate, and they are limited in duration due to their limited payload. Therefore, there is a current need for a flameless, passive, cost effective, multi-chemistry delivery method for spatial repellency that provides sustained and safe protection over weeks in order to increase adherence of use, and ultimately further reduce risk of vector-borne diseases. Controlled Release Devices (CRDs) were developed to overcome some of the limitations of current spatial repellent (SR) delivery systems.

**Fig 1:**
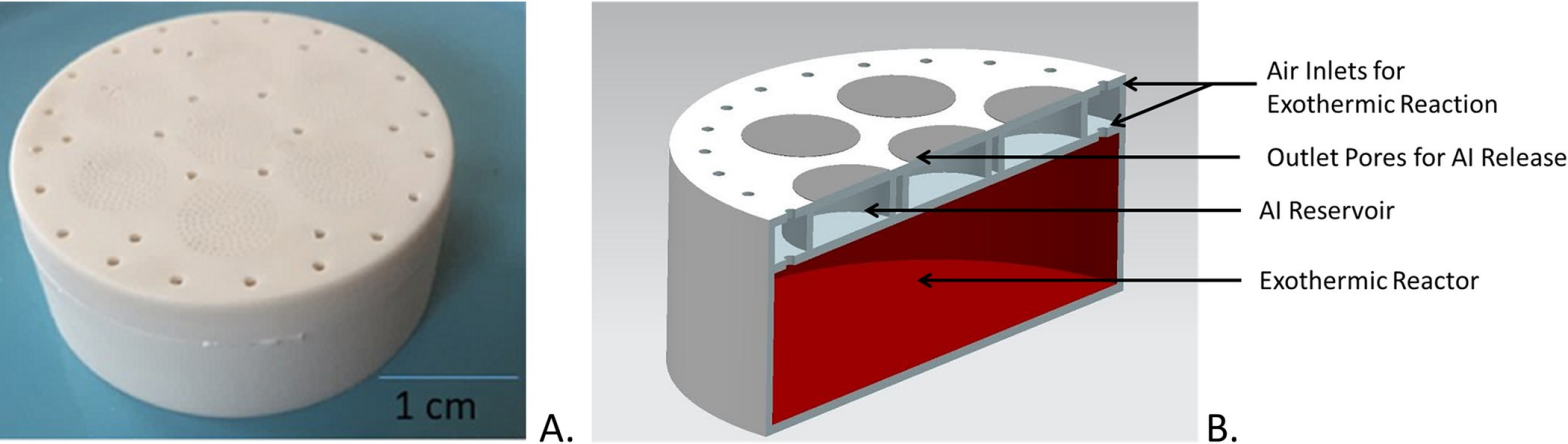
Controlled Release Device (CRD). A. Manufactured Device. B. Internal Components.

Present CRD design uses a set of wells where formulated AIs are placed, each well is capped by a lid that forms a chamber. The chamber lids integrate a set of tightly calibrated pores to control the release of the formulated AI. The CRD also includes an exothermic process that is activated when the device is open and exposed to air via inlets to provide an increase release rate. Fig 1A and 1B show an image of a CRD and its internal components.

The advantages of the CRD are: no electrical power, no battery; no open flame, making it safer than coils and candles; manufacturable with biodegradable materials, making it environmentally friendly; it can last up to two weeks; it can store multiple AIs. Present CRD design can be used in indoors or outdoors applications, and provides a cost-effective solution that can be mass produced and easily deployed.

To maximize CRDs performance, high volatility is required in the AI to be used while it has to be effective against Mosquitoes. As pyrethroids compounds are known to have high efficacy in protection against mosquitoes, metofluthrin was chosen [19]. Metofluthrin is commonly referred to as a Spatial Repellent (SR), but it is in fact considered an insecticide by the Environmental Protection Agency (EPA), which has oversight to approve new AIs in the US.

**Fig 2:**
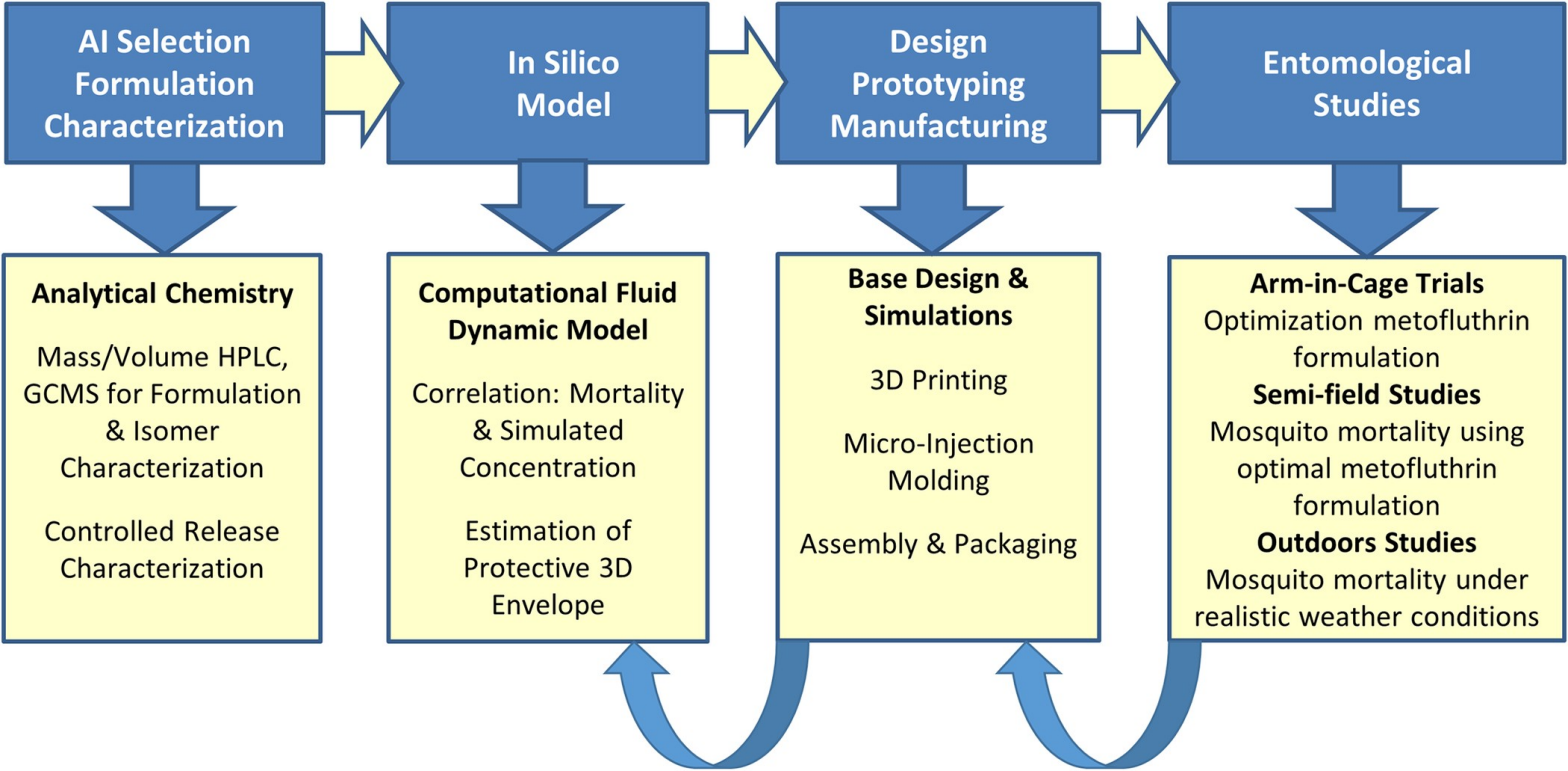
Process flow. Combination of experimental and numerical methods.

Herein we describe the systematic approach used in the CRD development, summarized in Fig 2. The first step was the physical characterization of several metofluthrin formulations to the evaporation rate of the AI. This data is then fed into a Computational Fluid Dynamics (CFD) model to predict estimated concentrations of AI in the space around the device. First generation CRDs were manufactured using 3D printing Stereo Lithography Apparatus (SLA) technology for rapid prototyping and eventually second generation CRDs were subsequently manufactured using micro-injection molding, a cost effective technique for mass production. CRDs were tested in an arm-in-cage study, performed to determine the optimal metofluthrin concentrations in a 15 minute trial. Knockdown and bite inhibition were used as entomological endpoints. Once the optimal metofluthrin formulation was determined, CRDs were tested with such formulation for semi-field studies in order to correlate the mortality with the AI concentration in space for 24 and 48 hour periods. Mortality was selected as the preferred entomological endpoint since it can be used to correlate estimated concentration with highly specific spatial locations for a given time point. The established correlation between mortality and estimated metofluthrin concentration can be used as a tool to further tailor the CRD design and form of deployment, e.g. number and distribution of CRDs, in order to target a protective volume with a desired mortality. Lastly, outdoor studies were performed to evaluate the performance of CRDs.

## Methods

Fig 2 shows a summary of the process flow detailing the used methodology.

### Physical characterization of AI release rates

Physical characterization of metofluthrin solutions was performed in order to characterize metofluthrin release rates. 50 mL falcon tubes containing metofluthrin with isopropyl alcohol (IPA) as a solvent and at the following concentrations: 1%, 5%, 10%, 30%, 50% (v/v), were left open for evaporation rate determination at room temperature conditions. IPA at 99% v/v, was used as solvent to increase metofluthrin volatility. IPA was chosen as solvent due to metofluthrin high solubility in it (313.2 gr/L), high volatility and low toxicity [19]. The total mass and total volume of remaining metofluthrin solution in each sample tube for 12, 24, 48 and 96 hours was measured using analytical balance and calibrated volumetric measurements under laboratory controlled temperature conditions (20–25°C). Changes in relative concentrations of metofluthrin and IPA were determined via Mass Spectroscopy (MS), estimating concentration of active isomers of metofluthrin E and Z. Using the same approach, the release rate of metofluthrin was determined. Water was estimated in the solution due to the hygroscopic nature of metofluthrin and IPA. Estimated metofluthrin release rates as a function of concentration were calculated from these studies. The rates were then scaled to the device geometry, calculated by multiplying by the ratio of the surface areas of the CRD to the falcon tube, in order to serve as input for the in-silico model to model spatial concentration evolution with time.

### CRDs design and manufacturing

CRDs preliminary versions were 3D printed, then manufactured using micro-injection molding in Zytel st801. CRDs design consisted of an exothermic reactor bottom containing a 50 mL reservoir to store an exothermic material (based on iron oxides) that acted as a heat source to increase volatilization of AI after activation and hence distribute AI in the volume to be protected faster. The device also included a middle reservoir array that included seven 0.5 mL reservoirs to store the AI. The device also included a top layer comprising a membrane with 30 inlet pores, each 1 mm in diameter, to allow oxygen diffusion into the exothermic reactor, and 1,000 outlet pores, each with 200 μm diameter pores for controlled release of the AI.

The exothermic reactor relied on oxygen to start the exothermic reaction, leading to a local increase in temperature of up to 55°C for eight hours and thereby enhancing the volatilization of the AI. Once cooled down, device mass rate was high enough to keep the protective volume until AI depletion.

Controlled release relies on evaporation of the AI formulation through the membrane pores until the AI reservoirs are depleted. The release rate of AI is dependent on the open surface area. Metofluthrin, an effective spatial repellent, was selected due its high efficacy, low toxicity profile and relatively high vapor pressure at room temperature [20]. CRDs were vacuum sealed, and an oxygen absorber was incorporated inside the package to further reduce the presence of any oxygen to prevent the activation of the exothermic reaction.

### Entomological studies

#### I. Arm-in-Cage studies

Arm-in-Cage studies were conducted at the Mosquito Fly Research Unit at USDA-ARS-CMAVE, Gainesville, Florida with human volunteers and the procedures followed those approved by the University of Florida Institutional Review Board (IRB-01, study protocol number 69–2006). All participants in the entomological studies were adults. The participants provided written informed consent for their participation. Experiments consisted on finding the optimal metofluthrin concentration to achieve maximum knockdown and bite inhibition. Metofluthrin formulations with IPA of 1, 5, 10, 30, 100% (v/v) and a combination of metofluthrin 30,100% (v/v) were evaluated. Two CRDs were placed in a small cage (50 cm x 50 cm x 50 cm) for 15 minutes prior to the start of the experiment. This induction time allowed for the exothermic reaction to initiate and the metofluthrin to diffuse in the cage space. Upon completion of the induction time, 100–120 previously preselected host-seeking female *Anopheles quadrimaculatus* mosquitoes, 6–8 days old, reared in a colony and received as pupae, were introduced in the small cage. A human volunteer introduced his arm for 15 minutes to allow mosquitoes to bite. Upon completion of each trial, device efficacy was evaluated by quantifying mosquito knockdown (number of incapacitated mosquitos on the cage floor or flying erratically), number of bites, and number of blood feds.

Six different metofluthrin formulations were tested with two CRDs per run, which included the following concentrations: 1%, 10%, 30%, 50%, 100% and an additional combination of 30% and 100%. Each experiment consisted of 8 replicates (N = 8), each of which was performed with a new pair of CRDs. One volunteer participated in the experiment. The experiments were performed over a four-week period using multiple batches of female Anopheles quadrimaculatus. Mosquitoes were 4–6 days old. Clean cages were dedicated to specific concentration experiment to prevent residual contamination. As control, same number of mosquitoes and no CRDs were used to confirm that the cages were not contaminated.

#### II. Semi-field Studies (SFS) in tents

To validate the results obtained in laboratory conditions, semi-field studies in standard military tents were conducted. Six CRDs using optimized metofluthrin concentrations selected from arm-in-cage studies were utilized. Three devices with metofluthrin 30% and three devices with metofluthrin 100% were placed at the center of a military tent, dimensions: 7 m (l) x 5 m (w) x 3 m (h). Pouches, each containing 20 *Anopheles quadrimaculatus* mosquitoes, were placed at three levels in nine different positions. The pouches were constructed of a grey fiberglass net material with very fine pores of approximately 500 μm. The pouches were built by cutting and sewing the screen into a shape of pyramid with approximately triangular, 15 cm side, and a height of 15 cm. Two fans were placed at the two opposite entrances of the tent, each providing an air flow speed of 1 m/s; the measured wind speed running across the tent was about 5 km/hr. The air flow provided a forced convection mechanism to improve the diffusion of metofluthrin to the tent air space. Experiments were conducted for 24 and 48 hours. Mosquito mortality on each pouch was accounted after each trial.

#### III. Outdoors studies

An outdoor study was conducted using *Anopheles quadrimaculatus* mosquitoes. Six CRDs with optimal metofluthrin concentrations (three devices with metofluthrin 30% and three devices with metofluthrin 100%) were used per replicate for this experiment (N = 6). 30 mosquito pouches were placed in two sets of three concentrically defined sets of rings located at 0.5 m, 1.25 m, and 2.5 m from the center (5 pouches per ring, 3 rings per set and 2 sets). The first set of rings was placed at 1 m from the ground, whereas the second one was placed at 1.5 m from the ground. CRDs were placed at the center of the rings, 0.5 m from the ground. Each pouch contained 20 mosquitoes. Five separate controls were used, each defined as a pouch each containing 20 mosquitoes at a distance of approximately 25 m away from the CRDs. The control pouches were placed 0.5 m from the ground, as follows: two pouches south, one pouch west, one pouch east, one pouch north. The control experiments were concurrently run with the rest of the experiments.

### Simulations

An in-silico model was developed to estimate metofluthrin spatial concentration distribution in a domain to match the observed mosquito mortality found during the entomological tests. The semi-field studies in tents configuration was chosen to reduce the air movement uncertainty that arises in an open domain. The domain consisted of an exterior volume where wind actual measured average speed and direction conditions were set. The tent was placed inside the domain with open doors to allow air to enter the interior domain. Fans present during the test were modelled inserting a speed jump at the fan location enforcing fan flow. Mass point sources were placed at the proper location with the measured device mass rate. A device is considered a point source due to its relative small size compared to tent volume. The model was developed using Computational Fluid Dynamics techniques in ANSYS [21] with k-ε turbulence for flow simulation and an implicit scalar transport scheme to tackle metofluthrin advection and diffusion in a transient solution. Changes in temperature do not affect significantly AI distribution, since natural convection dominates the mass transfer. From a fluid mechanics perspective, the estimated low AI concentrations will not lead to any buoyancy effect.

## Results

**Fig 3:**
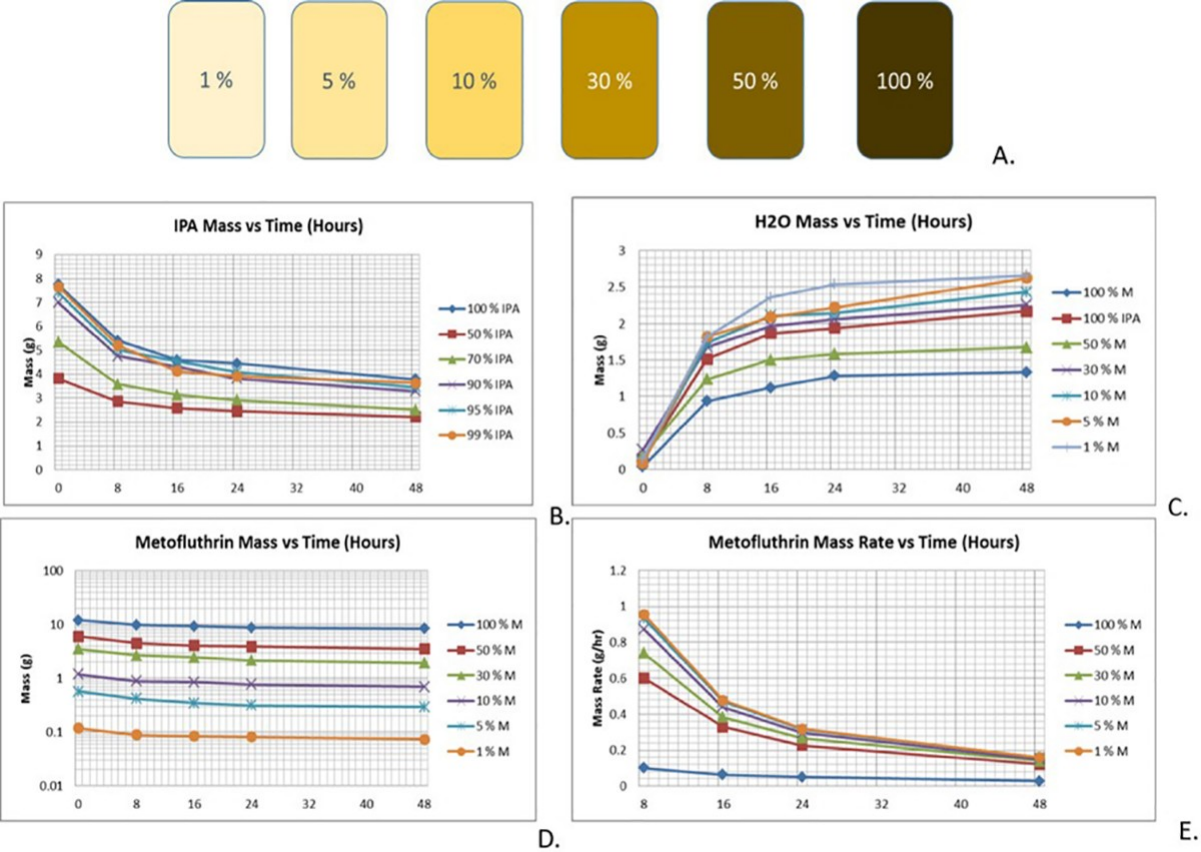
Characterization of formulations. Formulations of Metofluthrin/Isopropanol (IPA). Isomers analyzed using HPLC-MS. A. Tested formulations. B. IPA mass (in formulations) as a function of time. C. Water mass (in formulations) as a function of time. D. Metofluthrin mass as a function of time. E. Metofluthrin mass rate (in formulations) as a function of time.

To characterize metofluthrin release rates for each of the tested formulations, as shown in Fig 3A, metofluthrin (AI), IPA (solvent), and water mass were integrated in the calculations to determine the fraction of each of these components as a function of time. Fig 3B shows the mass of IPA as a function of time, Fig 3C shows the mass of water as a function of time, and Fig 3D shows the mass of metofluthrin as a function of time. Water content was studied together with IPA mass and metofluthrin mass due to its hygroscopicity.

IPA evaporation rate was similar for the proposed formulations, while metofluthrin increased its evaporation rate with the IPA fraction, which suggests that IPA increases the metofluthrin evaporation rate. Metofluthrin evaporation rate was calculated as the change in mass over time, shown in Fig 3E. In this plot, it is possible to observe that all curves converge towards the same rate level, which represents the 100% metofluthrin (beyond 48 hours).

**Fig 4:**
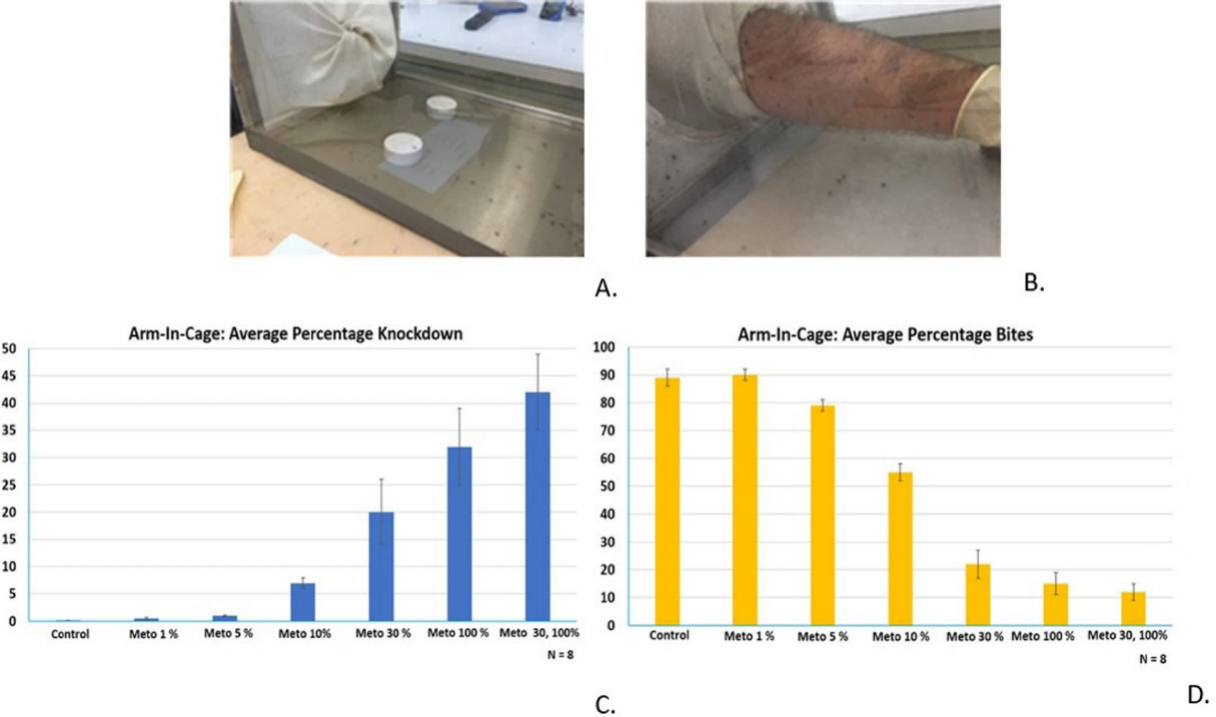
Arm-In-Cage trials. A. Small cage (50 cm x 50 cm x 50 cm) with two CRDs. B. Human volunteer exposing his arm inside a small cage. C. and D. Plots showing percentage of knockdown and percentage bites of CRDs at different metofluthrin concentrations. N = 8.

Selection of the optimal concentration to be used in the CRDs required the Arm-in-Cage studies as the first step. Experimental setup is shown in Fig 4A and 4B. Fig 4C shows the average percentage knockdown and Fig 4D shows the average percentage of mosquito bites as a function of concentration, ranging from 1% to 100% metofluthrin. It is possible to observe that knockdown increases with metofluthrin concentration while bites are reduced, with more data dispersion in knockdown than in bites.

A test with a combination of the two best performing concentrations was performed. One device with 30% metofluthrin and other one with 100% metofluthrin were tested. It was found that such combination performed even better than the best result obtained with two identical CRDs. This improvement may be attributed to the complementary of the different release kinetic profiles. One possible interpretation is that the first period of high evaporation was mainly driven by the 30% metofluthrin formulation, complemented by a second period of evaporation primarily driven by the 100% metofluthrin.

**Fig 5:**
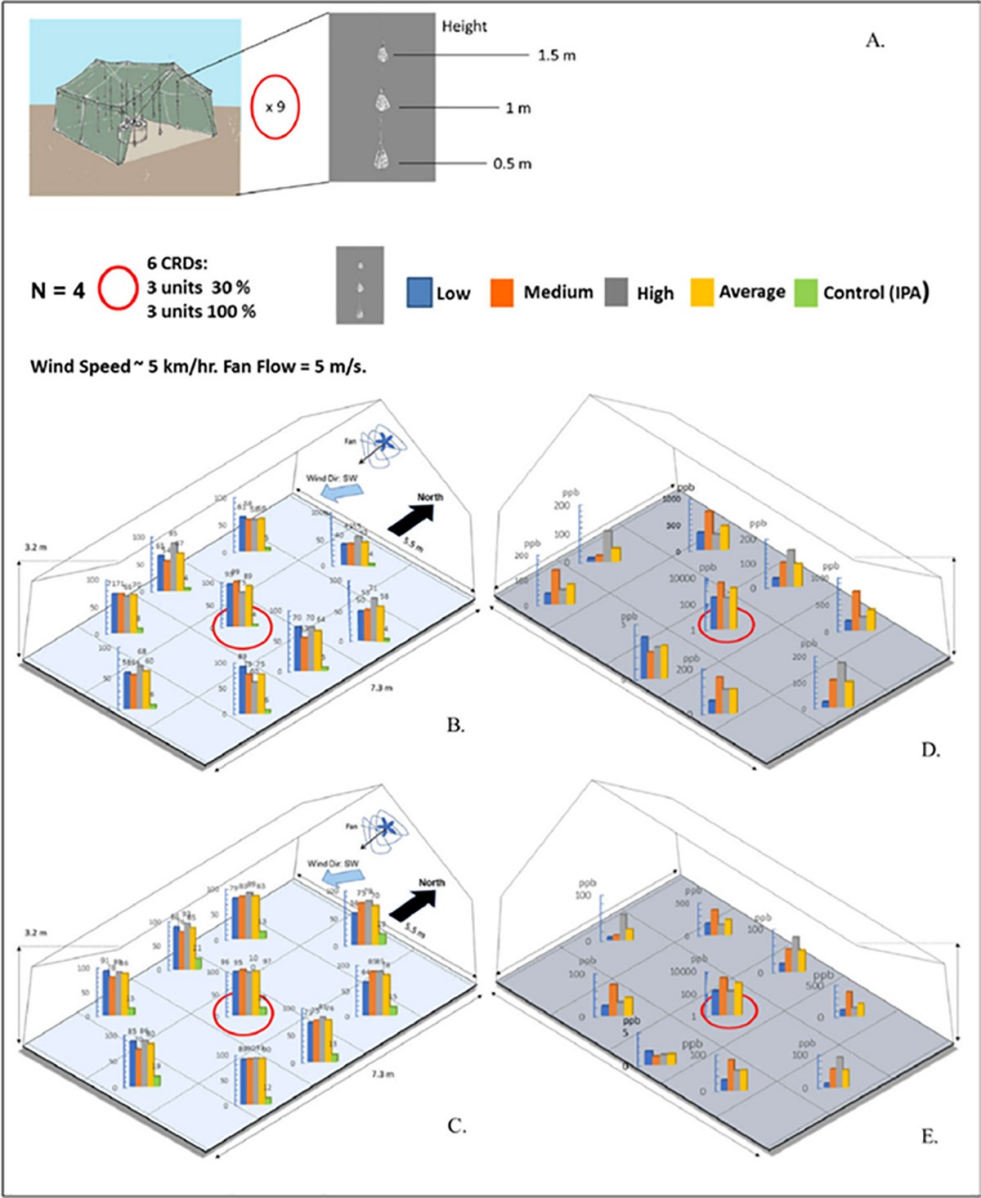
Semi-outdoor experiments. A. Experimental setup. B. C. Experimental results showing mosquito mortality per pouch per pouch location for 24 hours and 48 hours, respectively. D.E. Simulations results showing projected concentrations per pouch location for 24 and 48 hours, respectively.

Semi-field studies were carried out in tents after the optimization process performed in arm-in-cage studies. Fig 5A summarizes the experimental setup. Experimental results are shown in Fig 5B for 24 hours and in Fig 5C for 48 hours (N = 4). For each tent location a bar plot showing mosquito mortality at each of the three defined heights is shown, together with their average mortality per tent location (yellow) and the average control defined as a 100% IPA loaded CRD (green). A uniform mortality distribution is observed though locations and heights. A minimum mortality of 50% can be observed in the first 24 hours, reaching 75% in 48 hours, with no significant differences between locations and heights. From the CFD model, simulated metofluthrin concentrations at each location and height are plotted for 24 hours and 48 hours in Fig 5D and 5E, respectively.

**Fig 6:**
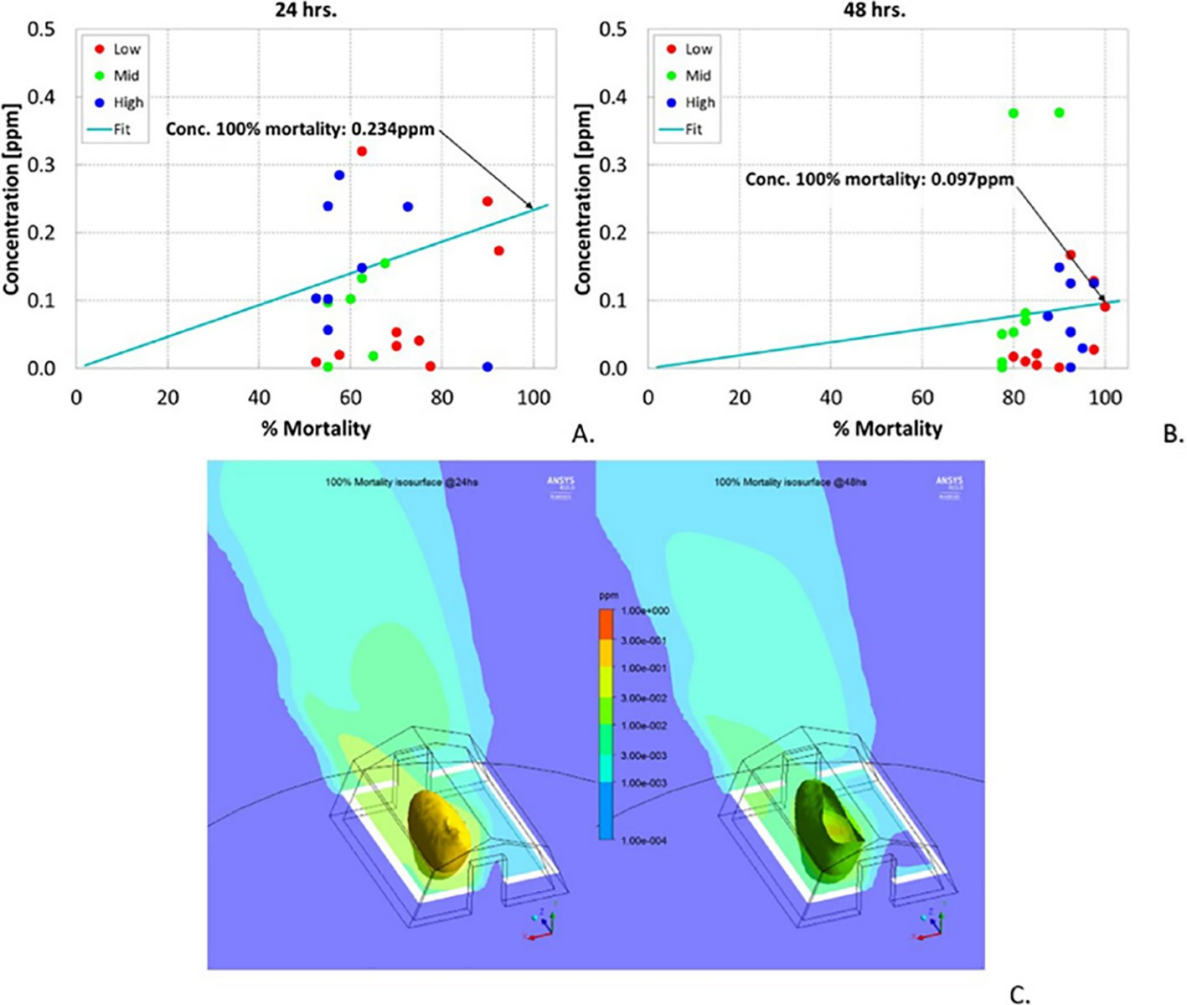
In Silico Model. Correlation between simulated concentrations (CRDs with 30, 100% metofluthrin) and mortality for mosquitoes inside pouches in tent. A. B. Correlations for 24, 48 h. C. Simulated ‘protection bubbles’ for achieving 100% mortality with boundary envelope concentrations of 0.234 ppm for 24 h with 0.224 mg/s release rate from 30% metofluthrin-based devices and 0.042 mg/s release rate from 100% metofluthrin-based devices (left frame); and 0.097 ppm for 48 h with 0.116 mg/s release rate from 30% metofluthrin-based devices and 0.024 mg/s release rate from 100% metofluthrin-based devices (right frame).

Furthermore, the estimated spatial concentrations obtained from the CFD simulations versus mortality for every location are plotted for 24 hours and 48 hours in Fig 6A and 6B, respectively. When analyzing this correlation plot, it was found that the lower and middle level pouches seem to require less concentration to reach the same mortality as the higher level pouches. Less concentration was required to be effective in 48 hours than in 24 hours, which could be attributed to the longer exposure of the mosquitoes to the metofluthrin.

Based on these plots, a linear regression analysis was performed to establish the correlation between mosquito mortality and metofluthrin concentration. The metofluthrin concentration for 100% mortality can therefore be calculated, resulting in 0.234 ppm for 24 hours and 0.097 ppm for 48 hours. Fig 6C shows the estimated iso-surface plots plotted for 24 and 48 hours. These iso-surfaces provide the spatial limit concentration found such that inside these convex surfaces a targeted mosquito mortality of 100% would be guaranteed.

**Fig 7:**
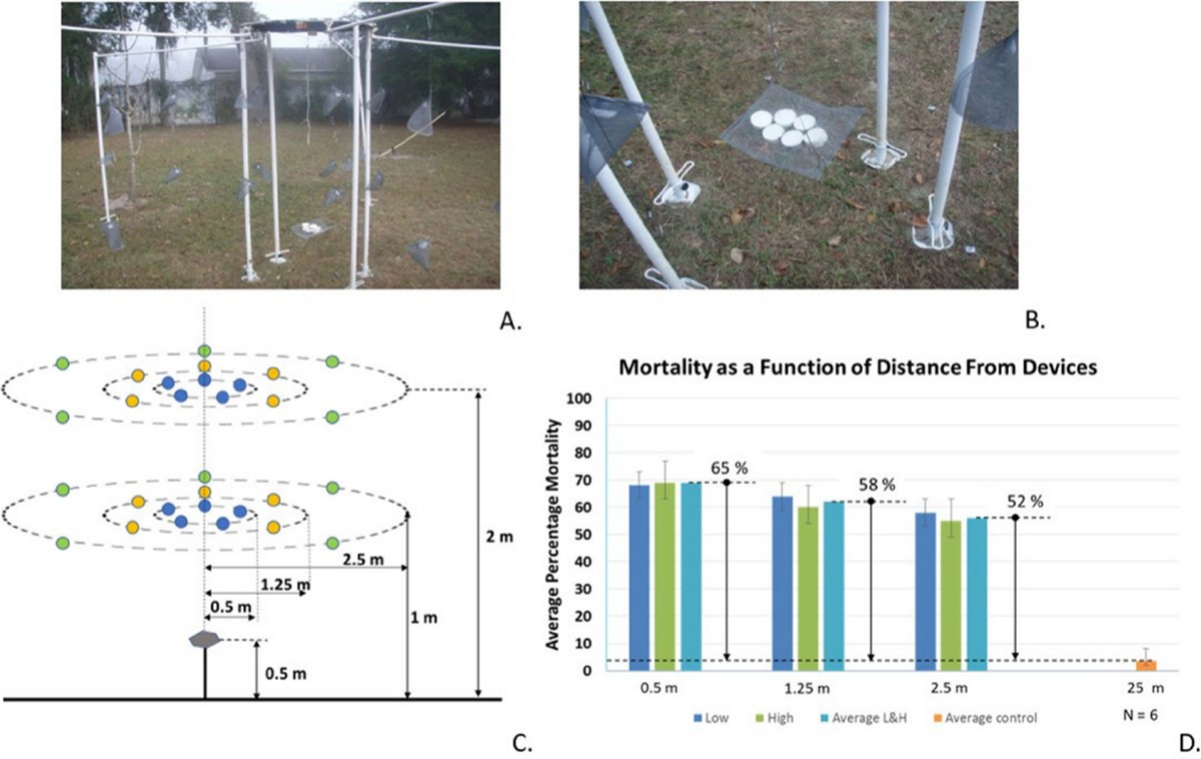
Outdoor experiments. A. and B. Experimental set up consisting of mosquito pouches placed at two levels (1 m, and 1.5 m from the floor) along three concentric rings (R = 0.5 m, 1.25 m, and 2.5 m). Six CRDs (three devices with metofluthrin 30% and three devices with metofluthrin 100%) were placed at the center, 0.5 m from the ground (N = 6). C. Diagram showing experimental setup. Colored dots represent pouches, each with 20 mosquitoes. Independent controls were placed approximately 25 m away from active devices. D. Plot showing average mosquito mortality per height and per radius for active devices compared to average mosquito mortality for the control.

Finally, Fig 7A and 7B shows the experimental set up for the outdoor experiments and Fig 7C the spatial location of the pouches. The bar chart plotted in Fig 7D shows the average mosquito mortality per pouch for the distances and heights evaluated from the CRDs. Results were plotted to show average mosquito mortality per level for a given distance from the source. Controls were also shown. A slight distance effect is observed showing a decrease in mortality with almost no dependence on pouch height. Mortality dispersion in not significant across directions, even considering the unrestricted air movement.

## Discussion

In silico modeling provides a powerful multi-dimensional tool to estimate AI concentration as a function of targeted mortality, for a desired 3D space in a given period of time and environmental conditions, which include 3D boundary conditions, temperature, and wind velocity. A correlation was established between simulated average concentrations and mosquito mortality obtained from semi-field experiments. Additional tests to address repellency and bite inhibition in open spaces, such as Human Landed Catches, could be performed in the future. Both parameters are associated with protective surfaces what makes them more complex to correlate with a spatial distribution.

The ability to correlate metofluthrin concentration with mosquito mortality in a 3D space as a function of time could potentially allow to customize SR delivery based on a target mortality and defined environmental conditions over a defined region. It is therefore possible to obtain predictive behavior of devices in terms of efficacy over a 3D space, defining a bubble of protection, over a period of time, while monitoring toxicity thresholds.

Arm-In-Cage trials demonstrated that formulations of metofluthrin with IPA at 30% and 100% provided the highest percentage of knockdown in the 40–50% range and bite inhibition in the 70–90% range. Semi-field studies were performed to show the performance of CRDs in a semi-outdoor environment. High mosquito mortality rates in the range of 60–90% relative to an independent control over a period of 24 and 48 hours validated the use of CRDs for protection against mosquito bites. It was also possible to plot the mortality (Fig 5B and 5C) per pouch, as well as simulations results for spatial concentration per pouch (Fig 5D and 5E) once the release rate for each device was empirically estimated. These projected concentrations allowed to estimate the performance of device as these values were correlated with the morality values. The established correlation provides a powerful tool for projecting device performance. Outdoor experiments provided another insight in the use of CRDs as a SR protection tool. Experimental results showed mosquito mortality in pouches in the range of 40–60% relative to an independent control over a period of 24 hours for distances of up to 2.5 m from the devices.

The presented design of CRD can accommodate volumes of up to 20 mL of AI, which represents approximately about half of the device total volume. Such a CRD version was designed to have an effective persistence (duration) of up to 2 weeks of continuous usage. The proposed cost of the device will be in the range of US$0.25–0.5 per unit for volumes of 1–10 million units. CRDs do not require electrical power, and do not constitute a fire hazard. Moreover, CRDs future material selections could include Mirel, a biodegradable polymer, or other selection of advanced polyhydroxyalkanoate polymers (PHAs), or even paper-based versions as an environmental friendly solution. Future device designs will include a small transparent indicator with a dye to show remaining device capacity.

The development and testing of a novel type of SR delivery system was introduced starting from idea conceptualization to formulation development, followed by *in silico* model, device design and manufacturing and entomological studies including arm-in-cage, semi-field and field experiments. CRDs were designed with a novel methodology that integrated combination of entomological endpoints and computational models to target efficacy and spatial protection as part of the design requirements. This multidisciplinary method allowed for a quantitative approach to device development and optimized performance. The design method allows for tailoring release kinetic profiles and field distributions for public health interventions in buildings, facilities and open areas. Spatial coverage can be achieved by the number and distribution of devices deployed. The duration can be tailored by the device capacity.

Experimental results have shown the potential use of CRDs as the next generation SR device. CRDs represent a simple and cost-effective solution for enhanced protection against vector-borne diseases.
